# Personality traits predict job stress, depression and anxiety among junior physicians

**DOI:** 10.1186/1472-6920-13-150

**Published:** 2013-11-09

**Authors:** Thomas Olsen Gramstad, Rolf Gjestad, Brit Haver

**Affiliations:** 1 Faculty of Medicine and Dentistry, University of Bergen, N-5020 Bergen, Norway; 2 Research department, Division of Psychiatry, Haukeland University Hospital, Bergen, Norway; 3 Department of Clinical Medicine, Division of Psychiatry, University of Bergen, Bergen, Norway; 4 Haukeland University Hospital, Bergen, Norway

**Keywords:** Junior physicians, Personality, Intervention studies, Medical students, Longitudinal study, Male, Female, Undergraduate medical education, Anxiety, Depression, Stress

## Abstract

**Background:**

High levels of stress and deteriorating mental health among medical students are commonly reported. In Bergen, Norway, we explored the impact of personality traits measured early in their curriculum on stress reactions and levels of depression and anxiety symptoms as junior physicians following graduation.

**Methods:**

Medical students (n = 201) from two classes participated in a study on personality traits and mental health early in the curriculum. A questionnaire measuring personality traits (Basic Character Inventory (BCI)) was used during their third undergraduate year. BCI assesses four personality traits: neuroticism, extroversion, conscientiousness and reality weakness. Questionnaires measuring mental health (Hospital Anxiety and Depression Scale (HADS) and Symptom Checklist 25 (SCL-25)), and stress (Perceived Medical School Stress (PMSS)) were used during their third and sixth undergraduate year. During postgraduate internship, Cooper’s Job Stress Questionnaire (CJSQ) was used to measure perceived job stress, while mental health and stress reactions were reassessed using HADS and SCL-25.

**Results:**

Extroversion had the highest mean value (5.11) among the total group of participants, while reality weakness had the lowest (1.51). Neuroticism and reality weakness were related to high levels of perceived job stress (neuroticism r = .19, reality weakness r = .17) as well as higher levels of anxiety symptoms (neuroticism r = .23, reality weakness r = .33) and symptoms of depression (neuroticism r = .21, reality weakness r = .36) during internship. Neuroticism indirectly predicted stress reactions and levels of depression and anxiety symptoms. These relations were mediated by perceived job stress, while reality weakness predicted these mental health measures directly. Extroversion, on the other hand, protected against symptoms of depression (r = −.20). Furthermore, females reported higher levels of job stress than males (difference = 7.52).

**Conclusions:**

Certain personality traits measured early in the course of medical school relates to mental health status as junior physicians during postgraduate internship training. This relation is mediated by high levels of perceived job stress.

## Background

Physicians have a high prevalence of mental health problems compared to other university educated professionals and the general population. They run an increased risk of suicide, and have a high prevalence of suicidal ideation and planning [1-7]. Several reports indicate that the time period during postgraduate internship is especially associated with increasing symptoms of depression [8-10]. Studies also indicate that female physicians are more at risk of developing mental disorders and have higher suicide rates than their male colleagues [3,11,12]. Suicide is the end of a process probably evolving over a long time, starting with common mental health problems. Important predictors of suicidal ideation, planning and attempts among doctors are high job stress levels combined with depression, female gender, living alone and personality traits such as reality weakness and neuroticism [6,13,14]. It is an enigma why doctors, who have extensive education, high socioeconomic status and the best life expectancies, would choose to end their own lives. Such socioeconomic factors usually protect against mental disorders, suicide, as well as general morbidity and mortality [15]. Further, mental health problems among physicians may affect their clinical performance and patient care.

These findings have led to international studies on mental health of medical students and their struggles throughout medical school [16-20]. Do specific factors related to the education, and later the practice of medicine itself, represent challenges not addressed in the medical curriculum and training? Which vulnerability factors in the individual medical student contribute to mental health problems? Finally, should a mandatory part of the medical curriculum include prevention of mental health deterioration among the students, thus preparing them for the strains experienced especially during their first years as doctors? These key questions have been widely investigated in studies both from Norwegian medical schools as well as internationally [18,21-25]. Reports indicate that medical school stress and factors related to the individual student, such as personality and previous mental health problems, contribute to mental health deterioration, and that intervention programs may reduce stress in medical school [22].

Studies show that medical students’ mental health is comparable to that of other students and the general population when enrolling at the university [26,27], but deteriorates during the study time [16,27,28]. Thus, the mental health of medical students is worse than other student groups’ during their final undergraduate years [28]. Symptoms of depression, perceived medical school stress, low age as well as personality characteristics are important factors for mental health deterioration among medical students and junior physicians, as shown by Tyssen and coworkers [18,21,29].

The personality trait neuroticism seemingly makes students more susceptible to medical school stress [21], increasing the risk of developing symptoms of depression [29]. Also at a general population level, neuroticism is a risk factor for developing symptoms of depression [30,31], as well as suicidal ideation and planning [30,32-35], while high levels of reality weakness predict suicidal ideation [34]. Extroversion, on the other hand, seemingly protects against suicidal thoughts at a population level [34].

Based on an ongoing study of future doctors educated from the University of Bergen, Norway, we explored which personality traits were associated with symptoms of anxiety and depression, and with reported job stress during their postgraduate internship training. Our aim was to investigate (1) which personality traits predict high levels of anxiety symptoms, symptoms of depression and job stress during internship; (2) if there is an association between reported stress levels during the final year of medical school and during internship; and (3) if there are any gender differences with regard to symptoms of anxiety and depression and job stress during internship. This investigation involved testing of both mediator and moderator models.

## Methods

### Participants and procedures

Two full classes of medical students (n = 281) at the University of Bergen, Norway participated in a mandatory intervention program during their third undergraduate year [36]. The participants were asked to fill in self-report questionnaires distributed by mail at baseline (T1) during their third undergraduate year, during their sixth undergraduate year (T2) and during their internship training following graduation (T3). Informed consent to participate was obtained from 83.3% (234/281) of the students.

At baseline 85.9% (201/234) of the students responded. The response rate at the last follow-up (T3) was 72.2% (169/234). The number of participants giving information on all the actual variables at the last follow-up was 149. Data from the third undergraduate year, the sixth undergraduate year and internship training are presented below.

Approval for this study was obtained from the Regional Committee for Medical Research Ethics and the Norwegian Social Science Data Service, which are the authority of research ethics in Norway.

### Questionnaires

#### Personality

Personality traits can be defined as stable characteristics of the individual’s personality [37,38], making up patterns of thinking, sensing and conduct. We used a 36-item version of the Basic Character Inventory (BCI) to measure personality traits among the students during their third undergraduate year of medical school (T1). Originally developed by Lazare et al. [39] and later modified by Torgersen [40], this instrument has been widely used in Norwegian mental health surveys [9,13,21,41]. BCI gives a thematic organization of four traits: neuroticism, extroversion, conscientiousness and reality weakness. A detailed description of these personality traits is given elsewhere [9,29]. Each trait is assessed by nine questions with a dichotomous response, so that every trait has a sum score between 0 (low) to 9 (high). Cronbach’s alpha coefficient was 0.67 for neuroticism, 0.61 for extroversion, 0.64 for conscientiousness and 0.59 for reality weakness at baseline.

### Perceived medical school stress and perceived job stress

Medical school stress levels were assessed during the third (T1) and sixth (T2) undergraduate year using the Perceived Medical School Stress (PMSS) questionnaire [42]. This instrument has undergone minor modifications for Norwegian medical school study conditions and is comprised of 13 items [43]. We used this modified 13-item PMSS version, in which each item has five response alternatives: Alternative 1 is “Totally disagree” and alternative 5 is “Totally agree”. PMSS is a recognized instrument which has proven useful to capture perceived stress in medical school [13,21,42]. The internal consistency reliability for PMSS in this study, measured by Cronbach’s alpha, was 0.79. In this study, we analyzed data from the PMSS recorded during the sixth undergraduate year (T2).

At the last follow-up (T3) however, the participants were physicians in postgraduate internship training. At this time, PMSS was therefore replaced by Cooper’s Job Stress Questionnaire (CJSQ), which records levels of perceived job stress [44]. We used a 32-item CJSQ version that has been adapted to fit postgraduate internship in Norway [9]. CJSQ has an acceptable reliability in Norwegian samples [9,45]. The internal consistency was Cronbach’s alpha = 0.92 for this scale. Perceived job stress was rated from 1 “Not at all” to 5 “Very much”. In order to compute a sum score, the response alternatives “Not an issue” and “Not at all” were coded 0.

### Stress reactions

Indicators of stress reactions were measured using Symptom Checklist 25 (SCL-25) [46], which is a shortened version of the original 90-item Hopkins Symptom Checklist (SCL-90) [47]. The SCL-25 is comprised of 25 questions regarding symptoms such as “Nervousness or shakiness inside” or “Worrying too much about things” during the past two weeks, and was applied on the participants during their third (T1) and sixth (T2) undergraduate year of medical school, as well as during internship training (T3). SCL-25 has a high reliability [46,48]. A shortened 5-item version (SCL-5) has previously been used in surveys of Norwegian medical students and physicians [41,49]. The stress reaction variable was constructed by selecting 13 items capturing typical stress related symptoms from the SCL-25 inventory for analyses. These items had mean values and variance indicating that they were of special importance to the participants in this study. The reliability of the selected items was Cronbach’s alpha = 0.88. The 13 items selected from the SCL-25 were: (1) headaches, (2) faintness or dizziness, (3) nervousness or shakiness inside, (4) feeling tense or keyed up, (5) feeling so restless you could not sit still, (6) feeling low in energy or slowed down, (7) blaming yourself for things, (8) crying easily, (9) trouble falling asleep, (10) feeling blue, (11) loss of sexual interest or pleasure, (12) worrying too much about things and (13) feeling everything is an effort. In this study, we analyzed the 13 selected items from the SCL-25 inventory assessed during internship training (T3).

### Symptoms of depression and anxiety

Originally constructed for patients admitted to somatic hospitals, the Hospital Anxiety and Depression Scale (HADS) records levels of depression and anxiety symptoms [50]. Seven statements assess levels of anxiety symptoms and seven statements assess levels of depression symptoms, both of which are scored on a four-point scale. The sum score of each subscale (levels of anxiety and depression symptoms) ranges from 0 to 21. The recommended cut-off score is ≥8 for each of the subscales HADS depression and HADS anxiety, indicating a possible major depression or clinical anxiety disorder [51,52]. HADS was applied during the third (T1) and sixth (T2) year of medical school and during internship (T3). Data from HADS recorded during postgraduate internship were analyzed in this study. During internship training, 10.8% of the participants scored above the cut-off score for the anxiety subscale, while 2.4% scored above the cut-off score for the depression subscale. The reliability of HADS has been found high [51,52]. In our sample, Cronbach’s alpha for HADS depression was 0.67 and for HADS anxiety 0.78 during internship.

#### Statistics

The analyses were descriptive statistics (mean value, standard deviation, skewness) and frequency distribution. The variables BCI, CJSQ, HADS and the previously defined 13 items from SCL-25 measuring stress reactions were skewed (skewness exceeding 1.0), and were therefore transformed using square root. In order to investigate reliability and to get information on validity we computed Cronbach’s alpha. Analyses of our research questions were done by correlation analyses, multiple regressions and structural equation models. Potential effects of gender were controlled for in the regression models. Most analyses were done using the Statistical Package for the Social Sciences (SPSS) version 19.0.0 software, while AMOS version 20 was used to analyze the structural equation models [53]. The model fit was evaluated by several fit indices: χ^2^ with significance test, Comparative Fit Index (CFI), Normed Fit Index (NFI), Non-Normed Fit Index (NNFI) and Root Mean Square Error of Approximation (RMSEA) with confidence intervals. The indices CFI, NFI and NNFI should be beyond 0.90, while RMSEA should be below 0.08 (fair fit) or preferably 0.05 (close fit) [54,55].

**Figure 1:**
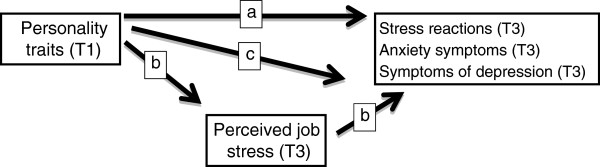
**Main models.** Illustration of the research problem with analyses of the relations between personality traits (T1), perceived job stress (T3) and stress reactions (T3), anxiety symptoms (T3) and symptoms of depression (T3) in a mediation model (*a* = direct effect, *b* = indirect effects). In addition, moderation effects were also tested (*c*). T1: During the third undergraduate year of medical school. T3: During postgraduate internship training.

Our investigation involved testing of both mediator and moderator models, as shown in Figure 1. In the mediation model, personality traits directly predict stress reactions, levels of anxiety and depression symptoms, and also indirectly through perceived job stress. A moderation model with testing of interaction effects, analyzes personality traits as moderators of the relationships between perceived job stress and the symptom dimensions anxiety, depression and stress reactions. The moderation model was analyzed using the SPSS regression module with a forced block-wise entry procedure. In step one the personality traits (BCI items) were entered, then perceived job stress (CJSQ items) in step two, and lastly the four interaction terms between personality traits and the job stress factor. However, the last block with interaction terms was entered as a forward procedure, allowing separate interaction terms to be statistically significant without being controlled for by the other interaction terms.

**Figure 2:**
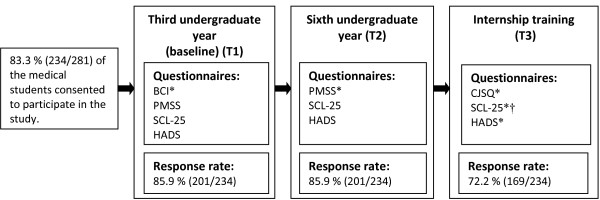
**Study phases.** Response rates and questionnaires used and analyzed in this study at different points of follow-up. BCI: Basic Character Inventory. PMSS: Perceived Medical School Stress. CJSQ: Cooper’s Job Stress Questionnaire. SCL-25: Symptom Checklist 25. HADS: Hospital Anxiety and Depression Scale. *Data from these questionnaires were analyzed in this study. †13 selected items from the SCL-25 inventory were analyzed.

Figure 2 gives an overview of the study phases with response rates and questionnaires at the different points of follow up.

## Results

### Descriptive analysis of sum scores

Descriptive analysis of sum scores is presented in Table 1. Extroversion had the highest mean value (5.11), while reality weakness had the lowest (1.51). Also, at a group level the participants scored higher on levels of anxiety symptoms (3.37) than symptoms of depression (1.48).

**Table 1:** Descriptive data

**Instrument**	**Dimensions**	**N**	**Mean value**	**Standard deviation**
HADS (T3)	HADS anxiety	167	3.37	3.02
	HADS depression	167	1.48	1.87
Personality traits, BCI (T1)	Neuroticism	191	3.71 (3.7)	2.10
	Extroversion	194	5.11 (5.2)	2.04
	Conscientiousness	191	3.38 (3.2)	2.11
	Reality weakness*	192	1.51*	1.57
CJSQ (T3)	Perceived job stress	169	29.25	18.94
SCL-25 (T3)	Stress reactions	168	7.38	6.57

The number of observations ranges from n = 167 to n = 194 due to missing data. The values in brackets are from a Norwegian nationwide survey on personality types among medical students conducted by Tyssen and coworkers [21]. *T1* During the third undergraduate year (baseline). *T3* During internship training. *HADS* Hospital Anxiety and Depression Scale. *BCI* Basic Character Inventory. *CJSQ* Cooper’s Job Stress Questionnaire. *SCL-25* Symptom Checklist 25. *Reality weakness was not included in the referred study.

### Correlation analyses

Table 2 shows a significant correlation between both neuroticism and reality weakness, and levels of anxiety and depression symptoms. These personality traits were correlated with high job stress levels and stress reactions. Extroversion was negatively related to symptoms of depression. No significant correlations between conscientiousness and the mental health variables were found. Further, perceived job stress was positively correlated with levels of anxiety symptoms, levels of depression symptoms and stress reactions. Gender had a significant prediction on job stress (r = −.19, p = 0.016), and female physicians experienced higher levels of job stress than males (female: 32.74; male: 25.22). In addition, perceived medical school stress during the sixth year of medical school was related to job stress levels during internship (r = .41, p = 0.000).

**Table 2:** Bivariate correlations

**Inventory**	**Items from inventory**	**Perceived job stress (CJSQ)**		**HADS depression**		**HADS anxiety**		**Stress reactions (SCL-25)**	
		**T3**		**T3**		**T3**		**T3**	
		**r**	**p**	**r**	**p**	**r**	**p**	**r**	**p**
Personality traits, BCI (T1)	Neuroticism	.19	.017	.21	.008	.23	.005	.31	.000
	Extroversion	-.04	.059	-.20	.011	-.15	.066	-.17	.149
	Conscientiousness	-.02	.079	.02	.768	-.01	.912	-.01	.884
	Reality weakness	.17	.004	.36	.000	.33	.000	.37	.000
CJSQ (T3)	Perceived job stress	1.00	.000	.35	.000	.41	.000	.55	.000

Correlation between personality traits (neuroticism, extroversion, conscientiousness and reality weakness measured at baseline), perceived job stress (CJSQ during internship), HADS depression (during internship), HADS anxiety (during internship) and stress reactions (13 selected SCL-25 items, during internship) among junior physicians graduated from the University of Bergen. *T1* During the third undergraduate year (baseline). *T3* During internship training. *HADS* Hospital Anxiety and Depression Scale. *BCI* Basic Character Inventory. *CJSQ* Cooper’s Job Stress Questionnaire. *SCL-25* Symptom Checklist 25.

### Prediction of stress reactions

**Figure 3:**
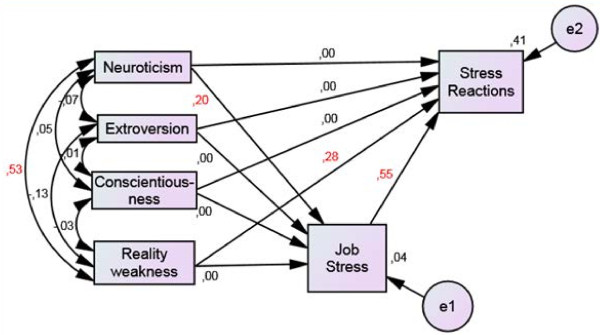
**Mediation analyses.** Mediation analyses between the personality traits neuroticism, extroversion, conscientiousness and reality weakness, perceived job stress and stress reactions (13 selected items from SCL-25). Arrows denote correlations. Correlations in red are statistically significant at .05 level. *e1* and *e2* are residual variances.

Figure 3 illustrates and gives the final estimates in the structural equation model. This model fitted data well (χ^2^ = 2.21, df = 7, p = 0.95, NFI = 0.98, NNFI = 1.08, RMSEA = .00, RMSEA_C.I._ = .000-.001, RMSEA_ϵ<.05_ = .98). Neuroticism predicted job stress, which in turn predicted stress reactions (13 selected SCL-25 items). However, there was no direct relationship between neuroticism and stress reactions, while this outcome was predicted by reality weakness. The explained variance of perceived job stress was 4%, while 41% of the variance in stress reactions was explained. A statistically significant correlation was found between neuroticism and reality weakness.

The relation between neuroticism and job stress was not statistically significant in the female sub sample (β = −.02), which implied no indirect effect from this personality trait on stress reactions. For males, on the other hand, this relationship was statistically significant (β = .36, p = 0.002). Reality weakness predicted stress reactions for both genders (females: β = .30, p = 0.001 and males: β = .35, p = 0.001). The relation between conscientiousness and stress reactions was statistically significant among males (β = .18, p = 0.028). Perceived job stress was related to stress reactions for both genders (β = .56, p = 0.001 for females and β = .40, p = 0.001 for males). Consequently, stress reactions during internship training were predicted by three variables (neuroticism, reality weakness, job stress) in males (explained variance 37%) and two variables (reality weakness, job stress) in females (explained variance 40%).

In a new model, perceived medical school stress (PMSS, analyzed from data collected during the sixth year of medical school) was weakly predicted by reality weakness (β = .04, p = 0.001), but stronger by neuroticism (β = .32, p = 0.001). Medical school stress levels predicted later job stress levels during internship (β = .53, p = 0.001). The relationship between job stress and stress reactions was almost as high (β = .50, p = 0.001). PMSS directly predicted the stress reaction score (13 selected SCL-25 items) (β = .11, p = 0.039). In this model, stress reactions were predicted by reality weakness (β = .22, p = 0.001). The job stress levels were not related to the personality traits (BCI), but were related to reality weakness and neuroticism through medical school stress. Gender differences in this model were not explored. This model achieved fair fit with data (RMSEA = .06); however, the RMSEA confidence interval indicated sampling instability (RMSEA_C.I._ = .000 - .111). Explained variance in the variables in this model was: medical school stress (PMSS) = 12%, perceived job stress (CJSQ) = 28% and stress reactions (13 selected SCL-25 items) = 40%. Compared to the earlier model, personality traits now predicted perceived medical school stress stronger than they did in their prediction of perceived job stress levels.

### HADS anxiety and depression

The BCI predictors and the mediating job stress variable were used in two new models with HADS anxiety and HADS depression as outcome variables. The results are presented in Table 3. The models fitted data within an acceptable level. Explained variance was 4% for the job stress variable, 33% for the anxiety variable and 22% for the depression variable. Additional analyses showed some gender differences. For males, perceived job stress was explained by neuroticism with 13% explained variance, while none of the personality traits predicted job stress levels among female physicians. Reality weakness predicted levels of anxiety and depression symptoms more strongly among males than females. Also, the relationship between neuroticism and perceived job stress only applied for males. Levels of depression symptoms were predicted by job stress levels and by reality weakness.

**Table 3:** Structural equation analyses

**Prediction of:**	**Perceived job stress (CJSQ)**			**HADS anxiety**			**HADS depression**		
**Sample:**	**Total**	**Females**	**Males**	**Total**	**Females**	**Males**	**Total**	**Females**	**Males**
Neuroticism	.20	0	.36	0	0	0	0	0	0
Extroversion	0	0	0	0	0	0	0	0	0
Conscientiousness	0	0	0	0	0	0	0	0	0
Reality weakness	0	0	0	.32	.25	.40	.28	.22	.43
CJSQ	-	-	-	.44	.52	.34	.34	.38	.24
R^2^	.04	.00	.13	.33	.33	.34	.22	.19	.29
Goodness of fit				MS^a^			MS^a^		
χ^2^, df, p-value				13.36	15	.58	10.22	15	.81
NFI				.90			.91		
NNFI				1.03			1.11		
RMSEA with C.I.^b^				.00	.000	.070	.00	.000	.050
RMSEA close fit				.85			.95		

Structural equation analyses of the perceived job stress, anxiety and depression variables. *HADS* Hospital Anxiety and Depression Scale. *CJSQ* Cooper’s Job Stress Questionnaire.

All reported parameter values are statistically significant at .05-level. Relationships given as zero indicate no statistical significant association.

MS^a^ Multi-sample analysis of females and males in one model.

C.I.^b^ Confidence interval with 95% low and high cut-off values.

No personality traits moderated the relationships between perceived job stress and the symptom dimensions anxiety, symptoms of depression or stress reactions.

## Discussion

The main finding of this study was that two personality traits predicted mental health problems among junior physicians. The trait reality weakness was related to higher levels of anxiety symptoms, symptoms of depression and stress reactions, while neuroticism only predicted stress reactions. Furthermore, the trait extroversion protected against symptoms of depression. These findings are on the whole in accordance with results from a series of similar studies among Norwegian medical students and later doctors [9,29,41], as well as findings from international studies [10,56]. Thus, these results are now quite robust.

Previous studies have investigated these vulnerability traits as well as protective traits predicting either mental health problems or perceived stress levels during medical school or internship. However, the present study investigates these two perceived stress variables as mediators of the relation between personality traits and mental health measures, and also whether the stress variables moderate these relationships. Applying structural equation analyses, we found that perceived medical school stress during the final year of medical school and later job stress during internship served as both predictors and mediators between personality traits, measured early in medical school, and mental health measures, assessed during internship. To our knowledge, this is the first paper describing a mediation effect from perceived stress. In this study, junior doctors with high scores on neuroticism experienced more job stress, and the same individuals had an increased risk of developing stress reactions. This suggests that doctors with a certain personality profile are more likely to develop mental health problems if they interpret environmental factors as more stressful than individuals with a more extrovert profile.

Research indicates that the strain experienced by students in medical school persist when they begin working as doctors during internship [9,18]. This also applied to the participants in this cohort, as students who were stressed during the graduating term reported higher stress levels also during internship. In addition, perceived stress in medical school directly predicted stress reactions among the junior doctors. This relation has also been established by Tyssen and coworkers, showing medical school stress to predict postgraduate mental health problems [18]. During internship, it seems that perceived job stress *per se* - not the actual objective stressors (long working hours, lack of sleep) themselves - contributes to mental health deterioration among junior physicians [9]. Thus, our findings indicate that a persistent high level of perceived stress is a variable of importance in identifying individuals at risk of developing mental health problems. It is also relevant for preventing future mental health deterioration. A previous intervention study from the University of Bergen has shown promising results in reducing perceived medical school stress [22], but it is still uncertain whether the intervention has a positive long-term effect on stress levels. Individuals with a personality profile making them especially vulnerable to medical school stressors are likely to profit from such intervention programs, in which they learn to cope with the many challenges they may face, thereby interpreting their environment as less stressful.

Gender differences with regard to perceived stress and mental health problems among medical students and physicians have been widely examined, however, findings are still discrepant. Some studies report that female medical students experience more mental health problems than males [10,17], while others fail to show any differences [9,18,29]. In this cohort, high neuroticism among females was not associated with higher levels of experienced job stress during internship, while the opposite was found for males. In addition, only male doctors who were above average conscientious reported stress reactions. Reality weakness, on the other hand, predicted stress reactions for both genders during internship.

This study and its design have several strengths and limitations. To our knowledge, this is the first study on personality traits and mental health among junior physicians in which mediation and moderation effects have been explored using structural equation modeling (SEM). Applying these methods of analyses allow for simultaneous testing of several relations, as well as testing of the effect of model improvements with chi-square difference tests of nested models. Another strength is the long follow-up period, spanning from the third undergraduate year to the first postgraduate year during internship, allowing for detection of time changes in the different mental health measures. The response rate at the last follow-up was 72.2%, which we consider an acceptable result.

One limitation in this study is that all data are based on self-report questionnaires. While these instruments have been validated for the Norwegian population, using self-report assessments may reduce the validity of the collected data, as the participants may have underreported their mental health problems. Further, as the personality traits of the respondents were assessed at baseline, one cannot rule out that their personality may have changed from baseline to follow-up. However, longitudinal studies indicate that personality does not change markedly during the course of life [57,58]. Also, the analyses in our survey are not controlled for age. In addition, some of the variables were skewed (skewness exceeding 1.0), and therefore transformation using square root was done. There was also a low range in the prevalence of measured personality traits, and this may have resulted in relatively low explained variance in the results.

## Conclusions

There is a relation between certain personality traits measured early in medical school and later mental health among junior physicians in internship training following graduation from the University of Bergen. Our findings are in keeping with previous studies on personality characteristics and mental health among medical students and junior doctors. Personality traits should be considered one of many potential vulnerability factors that constitute the foundation for mental health deterioration among junior physicians. Assessing personality traits may prove useful in detecting individuals at risk of developing mental health problems. Intervention programs aimed at reducing perceived stress and preventing future mental health problems should be further explored, especially in medical students with a vulnerable personality profile.

## Competing interests

The authors declare that they have no competing interests.

## Authors’ contributions

TOG drafted the manuscript and performed some statistical analyses. BH planned, designed and conceived the study, contributed to the interpretation the data and helped drafting and revising the manuscript. RG performed statistical analyses, contributed to the interpretation of the data and revised the manuscript. All authors read and approved the final manuscript.

## Pre-publication history

The pre-publication history for this paper can be accessed here:

http://www.biomedcentral.com/1472-6920/13/150/prepub
